# Cardiac magnetic resonance perfusion imaging using a single intravenous line

**DOI:** 10.1186/1532-429X-14-S1-P10

**Published:** 2012-02-01

**Authors:** Roshan Weerackody, Krishnaraj S Rathod, Steffen E Petersen, Saidi A Mohiddin, Ceri Davies, Mark Westwood

**Affiliations:** 1 London Chest Hospital, London, UK

## Background

CMR perfusion (CMRP) imaging using adenosine is increasingly used for the assessment of patients with known or suspected coronary artery disease. The scanning protocol requires administration of intravenous adenosine to achieve maximum hyperaemia followed by acquisition of CMR images during first pass perfusion of gadolinium. Traditionally this involves placing two intravenous lines, one in each arm, for the administration of adenosine and gadolinium. Two-way intravenous adapters allow administration of two compatible agents through a single intravenous line. We introduced this simple adapter for use during CMRP at our institution. We now report on our experience and the impact of this change to our CMRP protocol.

## Methods

We retrospectively analysed the CMRP scans performed before (January 2009 - December 2009) and after (January 2010 - December 2010) the introduction of the two-way adapter which was introduced in January 2010. First pass CMRP was performed on a Philips Achieva CV 1.5T MR scanner (Philips, The Netherlands), with a standardised acquisition protocol using an adenosine dose of 140µg/kg/min for 3 minutes. 3 short axis slices of 10mm thickness were acquired per cardiac cycle using a single shot prospectively gated balanced TFE sequence (TR 2.5ms, TE 1.3ms, Flip angle 500 and voxel size 2.8 x 2.8mm2) after the administration of a 0.1mmol/Kg bolus of intravenous Gadolinium. We analysed the patient demographics, scanning time, number of CMR operators to perform the scan and adverse events related to intravenous access.

## Results

4902 cardiac MRI studies were done between Jan 2009 and Dec 2010. The number of CMRP before and after the introduction of the intravenous adapter was 1485 (65.7%) and 1790 (67.8%) respectively (p=0.3, NS). There was no significant difference in age or gender between the two groups. However, the time taken complete CMRP was significantly shorter following the introduction of the two-way intravenous adapter (42.3±5.8min vs 37.3±8.1min, p<0.05). Additionally the number of operators required per scanning session was also reduced from three to two. The number of adverse events related to intravenous access site complications was low (0.1%) and there was difference between the two groups (3/1485 vs 1/1790, p=0.33).

## Conclusions

The introduction of a simple two-way intravenous adapter resulted in shorter scanning time, a reduction in the staffing requirements per session and no significant increase in adverse events. This highlights the importance of continuing adaptation in a busy CMR service and protocol optimisation which leads to potential financial savings and an improved patient experience.

## Funding

None.

